# The global burden of cervical cancer requiring surgery: database estimates

**DOI:** 10.1186/s13027-023-00562-3

**Published:** 2024-02-26

**Authors:** Emma R Allanson, Syed Nabeel Zafar, Chidinma P Anakwenze, Kathleen M Schmeler, Edward L Trimble, Surbhi Grover

**Affiliations:** 1 The Division of Obstetrics and Gynaecology, The University of Western Australia, Perth, WA Australia; 2 Department of Surgery, University of Wisconsin, Madison, WI USA; 3 Department of Radiation Oncology, The University of Texas MD Anderson Cancer Center, Houston, TX USA; 4 Department of Gynecologic Oncology & Reproductive Medicine, The University of Texas MD Anderson Cancer Center, Houston, TX USA; 5 National Cancer Institute, National Institutes of Health, Bethesda, MD USA; 6 Department of Radiation Oncology, Abramson Cancer Center, University of Pennsylvania, Philadelphia, PA USA; 7 Botswana-UPENN Partnership, Gaborone, GA Botswana

**Keywords:** Cervix cancer, Global, Low and middle income countries, Surgery

## Abstract

**Background:**

Scaling up surgical services for cervical cancer in low and middle income countries requires quantification of the need for those services. The aim of this study was to estimate the global burden of cervical cancer for which access to surgery is required.

**Methods:**

This was a retrospective analysis of publicly available data. Cervical cancer incidence was extracted for each country from the World Health Organization, International Agency for Research, Global Cancer Observatory. The proportion of cases requiring surgery was extrapolated from the United States Surveillance, Epidemiology and End-Result database. The need for cervical cancer surgery was tested against development indicators.

**Results:**

Data were available for 175 countries, representing 2.9 billion females aged 15 and over. There were approximately 566,911 women diagnosed with cervical cancer (95% CI 565,462–568,360). An estimated 56.9% of these women (322,686) would require surgery for diagnosis, treatment or palliation (95% CI 321,955 − 323,417). Cervical cancers for which surgery is required represent less than 1% of cancers in high income countries, and nearly 10% of cancers in low income countries.

**Conclusions:**

At least 300,000 cervical cancer cases worldwide require access to surgical services annually. Gathering data on available cervical cancer surgery services in LMIC are a critical next step.

## Background

More than 500,000 women are diagnosed each year with cervical cancer. The burden is greatest in low-and-middle income countries (LMICs), where it is the number one cause of cancer deaths for women in 42 countries [1]. Optimum management of cervical cancer requires a multidisciplinary approach including surgical, radiation oncology, medical oncology, pathology, imaging, and palliative care services [2]. Surgery plays an essential role in the diagnosis and management of cervical cancer care, and can often provide a cure for women with early stage disease [3]. However, while we know the burden of disease is highest in LMICs, we do not have a clear understanding on the met or unmet need of surgery. The capacity to deliver safe and appropriate management depends on the available surgical services and associated infrastructure. There is an imbalance between the data available on the need for and breadth of these services in high income countries, compared with LMICs, where the disease burden is greatest [4, 5].

The World Health Organization (WHO) has put out the call to eliminate cervical cancer by 2030 [6] which will require access to surgical services for diagnosis, treatment and palliation. Surgery for cervical cancer is primarily for curative intent and includes conization and/or simple hysterectomy for pre-invasive disease as well as radical hysterectomy with lymphadenectomy for early stage invasive cervical cancer. Simple or radical hysterectomy may also be performed following neoadjuvant chemotherapy and/or radiotherapy for locally advanced disease. Surgery is occasionally performed for recurrent disease with curative (pelvic exenteration) or palliative intent (colostomy, urinary diversion) [7]. There is a clear intersection between the countries where the burden of cervical cancer is greatest and those with the least ability to provide comprehensive surgical services [8]. Taking steps to scale up surgical services in LMICs requires quantification of the need for those services. Data registries regarding cervical cancer incidence, staging, treatment and outcomes in many countries are lacking, which limits the ability to assess the adequacy or lack thereof of existing surgical services in those same countries. The aim of this study therefore was to estimate the global burden of cervical cancer cases for which access to surgical intervention is required.

## Methods

This study was conducted in accordance with the STROBE-RECORD statement. Using publicly available data sources, we collated relevant cervical cancer data from 194 countries. We adopted the methodology used by Zafar et al. to estimate the global demand for cancer surgery, which has been described in detail elsewhere [8]. Briefly, the incidence of all cancers, and specifically cervical cancer incidence for the year 2018 was extracted for each country from the WHO and International Agency for Research factsheets on Cancer (IARC) and Global Cancer Observatory [9]. World Bank population estimates and economic data were used and countries were classified as high, upper middle, lower middle, or low income [10]. The Human Development Index (HDI) was obtained from United Nations Reports [11]. In order to estimate the number of cervical cancer cases requiring surgery, the United States Surveillance, Epidemiology and End-Result program (SEER) for the year 2013 was interrogated to establish the percentage of patients with a diagnosis of cervical cancer for which surgery was required [12]. Surgery was defined as any surgery with a diagnostic, therapeutic, palliative, or curative intent. The percentage of cases in the SEER database requiring this was 56.9%. This proportion was then multiplied by the incidence of cervical cancer for each country derived from the relevant fact sheets above. We recognised that the SEER data distribution of stages and therefore the number of cases requiring surgery will not be reflective of many LMIC, however given the comprehensive nature of the database, a pragmatic decision was made to apply these estimates.

Summary statistics (means and proportions) for the WHO geographic regions (Africa, Americas, South East Asia, European, Eastern Mediterranean, and Western Pacific) were analysed. The variation in cervical cancer cases by region were calculated, as were low, middle and high tertiles for each region for absolulte number of cases. The relationship between cervical cancer cases requiring surgery and HDI class and income classification were analysed with a Chi-square test. All p values were two-sided. All data were analysed using SAS Studio (SAS Institute Inc., Cary, NC, USA).

## Results

Data were available for 175 of the 194 countries, representing 7.6 billion people, and 2.9 billion females aged 15 and over. Data were aggregated and presented by WHO geographic region. Among the 2.9 billion females aged 15 years and over, there were approximately 566,911 cervical cancer cases (95% CI 565,462–568,360). Based on the SEER estimates, this included 322,686 requiring surgery for diagnosis, treatment or palliation at some point in their care. Data were aggregated and presented by WHO geographic region. Overall, cervical cancers represent 15% of the burden of all cancers in the African region (range 3.3–38%), and 1.7% of all cancers in the European region (range 0.5–10.1%). This equates to a more than 9-fold difference in the number of cervical cancer cases requiring surgery as a function of all cancer cases (9.08% in the African region compared to 0.97% in the European region) (Table 1). The low, middle and high tertiles for number of cases (absolute values) for each region are as follows; African (430, 2362, 14,080), Americas (468, 1518, 16,298, South East Asian (1389, 7535, 96,921), European (337, 791, 18,163), Eastern Mediterranean (149, 444, 5601), Western Pacific (276, 1243, 106,429).

**Table 1:** Variation in female population, cancers, and cervical cancers by WHO region

Region	Countries (n)	Female population 15 and over (millions)	Total cancers (thousands)	Total cervical cancers	Cervical cancers as a % of total cancers	Cervical cancers needing surgical intervention (% of total cancers)
African	45	306	699 (95% CI 697–701)	111,468 (95% CI 110,868–112,068)	15.94	63,448 (9.08)
Americas	30	398	3036 (95% CI 3032–3039)	71,212 (95% CI 70,695–71,729)	2.35	40,534 (1.34)
South East Asian	11	714	1793 (95% CI 1791–1796)	158,692 (95% CI 157,947–159,437)	8.85	90,327 (5.04)
European	50	395	4064 (95% CI 4060 − 4048)	69,058 (95% CI 68,547–69,569)	1.70	39,308 (0.97)
Eastern Mediterranean	21	225	585 (95% CI 585–587)	15,865 (95% CI 15,621–16,109)	2.71	9030 (1.54)
Western Pacific	18	871	5764 (95% CI 5760–5769)	140,616 (95% CI 139,890–141,342)	2.44	80,039 (1.39)
Total		2,908	15,942 (95% CI 15,934–15,950)	566,911 (95% CI 565,462–568,360)		322,686 (2.02)

The proportion of cervical cancers as a component of total cancers as well as the number cervical cancers requiring surgery, both separated by HDI and income status are shown in Table 2. Cervical cancers for which surgery is required represent less than 1% of cancers in high income countries or very high HDI class countries, and nearly 10% in low HDI class or low income countries.

**Table 2:** Cervical cancers in need of surgery by HDI class and country income classification

	Total cancers (thousands)	Total cervical cancers (% total cancers)	Cervical cancers needing surgical intervention (% of total cancers)	p-value
HDI Class				
Very high	6767	91,010 (1.34%)(95% CI 90,423–91,597)	51,803 (0.77)	< 0.0001
High	6082	180,570 (2.97%)(95% CI 179,750–181,390)	102,780 (1.69)	
Medium	2477	206,398 (8.33%)(95% CI 205,545–207,250)	117,482 (4.74)	
Low	530	84,227 (15.9%)(95% CI 83,705–84,749)	47,942 (9.04)	
Income classification				
High	6084	65,520 (1.08%)(95% CI 65,021–66,019)	37,294 0.61)	< 0.0001
Upper middle	6686	215,252 (3.22%)(95% CI 214,357–216,147)	122,521 (1.83)	
Lower middle	2730	217,925 (7.98%)(95% CI 217,047–218,802)	124,043 (4.54)	
Low	418	66,419 (15.89%)(95% CI 65,958–66,882)	37,806 (9.05)	

## Discussion

### Principal findings

We estimate that at least 300,000 women require surgery for cervical cancer every year worldwide, half of which occur in lower middle and low income countries. In the South East Asian and African regions, the number of cervical cancer cases estimated to require surgery represent 5% and 9% respectively of all cancer cases. This is in stark contrast to the European region, where only 0.97% of all cancers are cervical cancer cases requiring surgery.

## Results

While assessments of available surgical services for all cancers are reported as a part of the WHO plan for the control of non-communicable diseases [5], there are less data available on the specific surgical services for the management of cervical cancer. With a fifth of the cervical cancer cases estimated here occurring in the Africa region, it is clear that detailed registries of both cases (with accurate staging data) and available surgical services are critical to improving surgical care [13]. WHO estimates of the overall global surgical workforce identifies critical shortages, with particular disparities in access between high and low income countries [14]. From a cancer specific point of view, Zafar and colleagues estimated the cancer patient to surgeon ratio (number of patients in need of cancer surgery per surgeon) for all cancers globally. The median ratio ranges from 7.32 patients in need of cancer surgery per surgeon per year in the European region to 80 in the African region [8]. With cervical cancer making up 10% of the surgical burden in the African region it very likely that there are not enough surgeons to care for the women with cervical cancer requiring surgery.

### Clinical implications

There are known deficiencies in available facilities and a lack of surgeons trained in gynecologic oncology in LMICs, particularly in Sub-Saharan Africa, as a consequence of broader health system limitations, as well as access to adequate training in many geographic regions [15, 16]. To meet the global demand for cervical cancer surgery we will need innovative ways to increase the gynaecologic oncology surgical workforce in addition to enhancing screening and preventative programs. Steps that have already been taken to achieve this include Project ECHO (Extension for Community Healthcare Outcomes), a telementoring program providing training and support for clinicians managing cervical cancer in LMICs [17]. As well as this, and in order to meet the need for a strengthened surgical work force in LMICs, the International Gynecologic Cancer Society (IGCS) have since 2017 partnered clinicians from low resource regions with hospitals in high income settings to formally train and mentor them in gynaecologic oncology [18]. Efforts to strengthen training for gynaecologic cancer surgery should be integrated into national cancer control and national surgical plans, as well as parallel efforts to build capacity for imaging, pathology and laboratory medicine, anesthesia, nursing, ICU care, post-operative care, and the relevant supply chains.

The burden of cervical cancer cases will continue to be intricately linked with the presence of cervical cancer prevention programs (both HPV immunization and cervical screening). While a majority of countries globally report the existence of a cervical screening program, it is apparent in many settings that there is limited coverage of screening relevant populations despite this [6]. This, in combination with a population of women who have not been the targets of HPV immunization programs (as a function of age), means that there will be an ongoing and significant need for adequate surgical services for cervical cancer.

### Research implications

Cervical cancer specific data registries, as well as detailed information regarding available cervical cancer surgery services are a critical next step.

Access to safe surgical care has traditionally not been a major focus of public health efforts [4], however this must be bought to the forefront alongside cervical cancer prevention programs if we are to achieve the goal of cervical cancer elimination.

### Strength and limitations

This analysis gives a comprehensive overview of the need for surgery for cervix cancer management globally. It is possible that the number of cases requiring surgical intervention is overestimated for some regions presented here. The number of cases requiring surgery is estimated from cases in the SEER database and this may not be reflective of the distribution of cases requiring surgery in all settings. Women in the United States of America (USA) are more likely to present with early stage disease and undergo treatment with surgery, where as those in lower income settings are more likely to present with advanced stage disease requiring radiotherapy, chemotherapy or palliative care [19]. While we made a pragmatic decision to use the SEER data, some of the potential overestimate in cases requiring surgery in doing this may be offset by the increased likelihood of surgical treatment in lower income countries for more advanced stages of disease when compared to the USA [20]. This is primarily due to a lack of radiotherapy resulting in patients undergoing primary surgery for larger tumours and/or administration of neoadjuvant chemotherapy followed by surgery. Accepting variations across regions (e.g. approximately 2/3 of cervical cancer cases in Sub-Saharan Africa are stage 3–4 at diagnosis [21]), modelling estimates a distribution of disease in six LMIC regions to be stage 1 in 8–34% and stage 2 in 19–43% of cases [22], suggesting that despite the potential for overestimate, there remains a large number of cases for which surgery is indicated. Furthermore, with increasing access to HPV immunisation and screening, there should ideally be a shift over time in the distribution of cases in LMICs such that the SEER estimates increasingly reflect what is needed in those settings.

## Conclusions

We estimate at least 300,000 cervical cancer cases worldwide require access to surgical services annually and the variation in available cervical cancer prevention programs across regions will continue to contribute to this significant global demand for cervical cancer surgery.

## Data Availability

The datasets generated and/or analysed during the current study are available in the World Health Organization. World Population Fact Sheets repository, https://gco.iarc.fr/today/fact-sheets-populations?population=900&sex=0 in the orld Bank Group. World Bank Country and Lending Groups repository, https://datahelpdesk.worldbank.org/knowledgebase/articles/906519-world-bank-country-and-lending-groups. and in the National Cancer Institute. Surveillance, Epidemiology and End Results Program repository, https://seer.cancer.gov/.
